# Mitigating exhalation puffs during oxygen therapy for respiratory disease

**DOI:** 10.1063/5.0057227

**Published:** 2021-08-05

**Authors:** Arshad Kudrolli, Brian Chang, Jade Consalvi, Anton Deti, Christopher Frechette, Helen Scoville, Geoffrey R. Sheinfeld, William T. McGee

**Affiliations:** 1 Department of Physics, Clark University, Worcester, Massachusetts 01610, USA; 2 University of Massachusetts Medical School-Baystate, Springfield, Massachusetts 01107, USA; 3 PO Box 459, Andover, Massachusetts 01810, USA

## Abstract

We investigate the dispersal of exhalations corresponding to a patient experiencing shortness of breath while being treated for a respiratory disease with oxygen therapy. Respiration through a nasal cannula and a simple O_2_ mask is studied using a supine manikin equipped with a controllable mechanical lung by measuring aerosol density and flow with direct imaging. Exhalation puffs are observed to travel 0.35 ± 0.02 m upward while wearing a nasal cannula, and 0.29 ± 0.02 m laterally through a simple O_2_ mask, posing a higher direct exposure risk to caregivers. The aerosol-laden air flows were found to concentrate in narrow conical regions through both devices at several times their concentration level compared with a uniform spreading at the same distance. We test a mitigation strategy by placing a surgical mask loosely over the tested devices. The mask is demonstrated to alleviate exposure by deflecting the exhalations from being launched directly above a supine patient. The surgical mask is found to essentially eliminate the concentrated aerosol regions above the patient over the entire oxygenation rates used in treatment in both devices.

## I. INTRODUCTION

Oxygen therapy is the major treatment modality for patients with COVID-19 and other respiratory diseases who have low blood oxygen levels.^1,2^ Depending on the severity of the condition, supplementary oxygen is delivered with various devices, including a nasal cannula or a simple O_2_ mask at the primary level, and more intensive treatment with high flow nasal cannula and the continuous positive airway pressure therapy, and finally, at the most advanced level, with full ventilatory support with endotracheal intubation.^1^ While more intensive methods are administered in highly controlled clinical settings, the nasal cannula and the simple O_2_ mask are used all over the world not only in clinical settings but also at home and assisted care facilities. Further, these primary devices are used to provide supplementary oxygen during transportation to healthcare points, in waiting areas, and other improvised locations when healthcare support is stressed, such as during the current COVID-19 pandemic.

It is well known that the exhalations from subjects with respiratory disease can carry virus-laden aerosols which may infect healthy individuals depending on their distance and duration of exposure.^3^ A typical adult exhales on average 5–6 l of air per minute while breathing normally with a tidal volume of approximately 500 ml at 12 breaths per minute, or with a lower tidal volume and higher compensatory breaths per minute while experiencing shortness of breath.^4,5^ Pressurized tanks are used to deliver oxygen through an oxygenation device at a similar, if not higher, flow rate. Such noninvasive treatments, however, raise the possibility of greater dispersal distances of infected aerosols, which increases the risk to caregivers due to the corresponding increase in air flow near the patient.

Indeed, bioaerosol-laden exhalations through high flow oxygenation devices and their impact on infectious disease spread have been studied^6,7^ and informed early strategies used in treating COVID-19 patients by intubation because it allows sealed air pathways and minimal exhalation dispersal in the vicinity of the patient.^8^ Poor outcomes following intubation have driven patient care to less invasive oxygen therapy but by protecting clinicians and other caregivers with personal protection equipment (PPE) to minimize aerosol inhalation with N95 and medical masks.^8,9^ Clinically, it is assumed that surgical face masks suffice for the prevention of viral transmission from respiratory droplets, while N95 respirators provide additional protection from airborne transmission via bioaerosols.^10,11^

A significant body of work is being developed by analyzing the dispersal of aerosols and larger droplets over a range of exhalations resulting from activities such as talking, singing, sneezing, or coughing,^12–15^ and mask mitigation to reduce exposure.^16–18^ Several recent studies have utilized computational fluid dynamics to explore exhalations in terms of more detailed flow structures and complex scenarios.^19–22^ Nonetheless, there is still a poor understanding of the physical characterization of exhalation dispersal while breathing under shortness of breath conditions, as experienced by infected patients. This understanding is still necessary because infections to healthcare workers remain a significant concern^23,24^ during the current pandemic where healthcare facilities and PPEs are less than optimal, particularly with the new infectious variants of the SARS-CoV-2 virus. Indeed, COVID-19—along with severe acute respiratory syndrome (SARS), influenza A (H1N1), Middle East respiratory syndrome (MERS), and ebola—is one of five major respiratory infectious diseases to emerge in just the last 20 years alone.

Exhalation dispersal mitigation by a surgical mask over high flow oxygen therapy has been proposed,^25^ and preliminary clinical data are available for mitigation of aerosols close to the patient where bedside care is delivered.^26,27^ The relative placement of a mask in the case of a patient receiving supplemental oxygen has been recently discussed,^28,29^ but a clear demonstration of mask efficacy when worn by patients using commonly used oxygen delivery devices remains unclear and is not practiced widely.^30^ Exhalation puffs—periodic turbulent exhalations with significant linear momentum and energy^13,31^—emerging from masks with vents are documented,^16^ but hospitalized COVID-19 patients receiving oxygen rarely use further mitigation strategies. Evidence for various comparisons about masks used in healthcare settings and the associated risk for COVID-19 remains insufficient.^32^ Any added risk posed by patients while being treated with a nasal cannula and simple O_2_ mask remains less appreciated.

In this study, we investigate the exhalations corresponding to a simulated patient being treated with a primary oxygenation device. The dispersal volume and density of the exhaled aerosol are visualized and characterized over space and time. We then demonstrate that a loosely placed surgical mask over a nasal cannula, or simple O_2_ mask, decreases and redirects exhalations downward, and thus away from the faces of caregivers. The surgical mask is only loosely placed in our mitigation strategy to alleviate any concern for increased work of breathing.

## II. METHODS

**FIG. 1.**
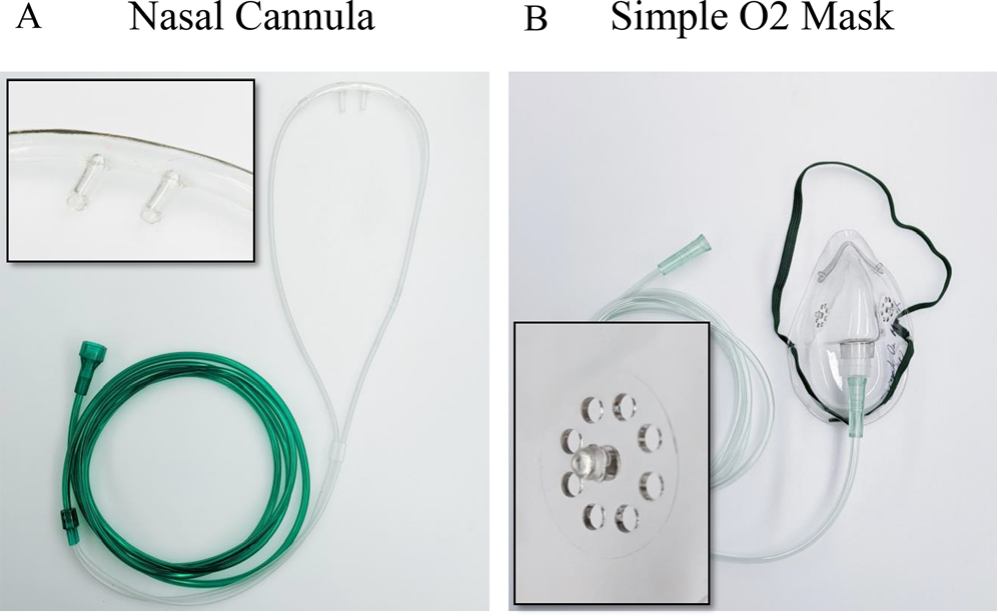
Images of the oxygenation devices used in the study [(a) and (b)]. The nasal cannula is a tube that splits into two prongs that are partially inserted into the nose. The simple O_2_ mask is a mask that covers both the nose and mouth with a tube that delivers oxygen. Insets show closeup of the prongs in the nasal cannula (a) and the ventilation holes in the simple O_2_ mask (b).

We examine two commonly used oxygenation devices: the nasal cannula and the simple O_2_ mask, shown in Fig. 1. The nasal cannula is a tube that splits into two prongs that are partially inserted into the nose. Oxygen is then delivered through the nose at a prescribed flow rate *Q* ranging between 2 lpm and 8 lpm (see Table I). The simple O_2_ mask covers both the nose and mouth of the patient but has two vents that would allow air to freely pass in and out of the mask. Meanwhile, a steady oxygen supply is delivered through the mask at a rate of *Q* ranging between 4 lpm and 12 lpm. Such values are commonly used in practice for patients undergoing respiratory therapy.^5^

**TABLE I.** Summary of the oxygenation devices, flow rates in liters per minute (lpm), and breathing tidal cycles in breaths per minute (bpm) investigated (*Q* = 0 lpm cases were conducted for calibration purposes).

Oxygenation device	Flow rate *Q* (lpm)	Tidal volume *V_t_* (ml)	Rate *f* (bpm)
None	⋯	350	20
Nasal cannula	0, 2, 4, 8	350	20
Simple O_2_	0, 4, 8, 12	350	20
Nasal cannula	2	500	12
Simple O_2_	4	500	12

**FIG. 2.**
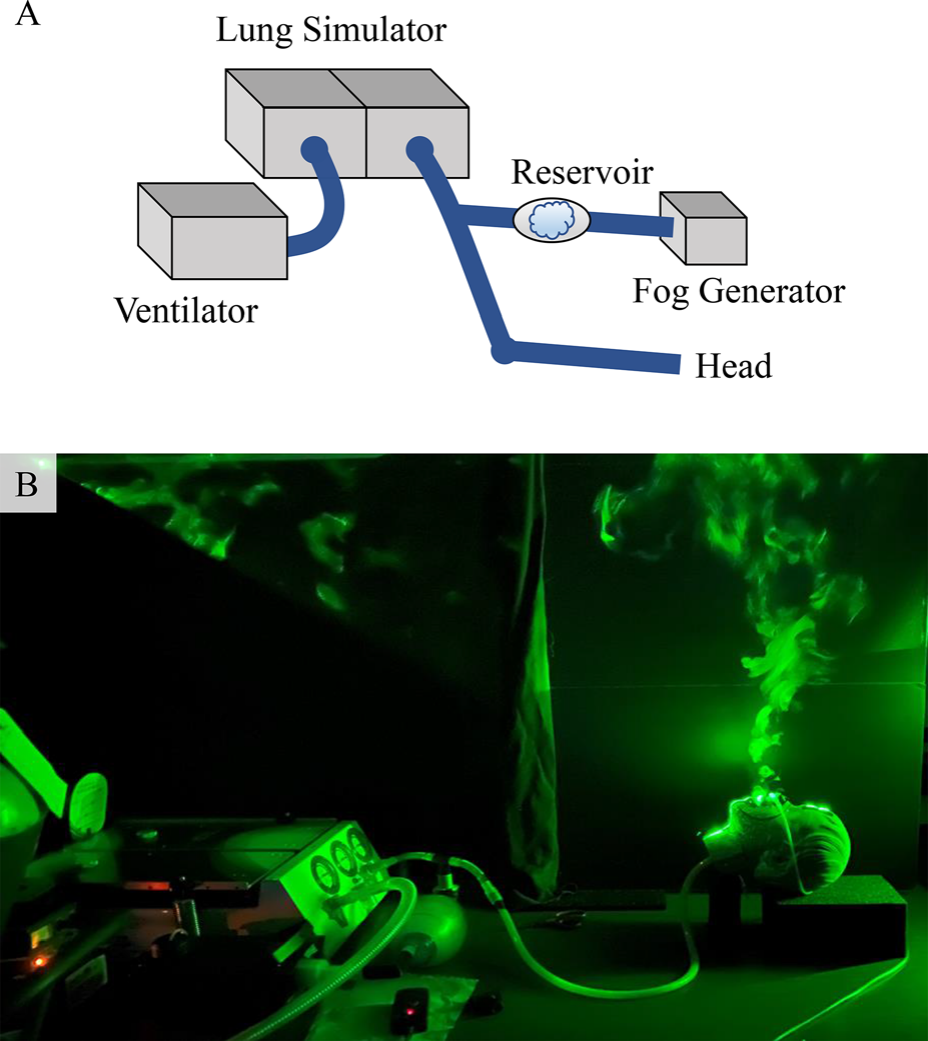
(a) The study apparatus consists of a dual lung simulator driven by a ventilator coupled with a second lung chamber which “breathes” air through the manikin head shown with a prescribed frequency and tidal volume. (b) Image of system with laser-sheet lighting used for aerosol visualization. The exhalations are visualized using water–glycerol aerosols (fog) released through the manikin mouth and/or nose during exhalation.

We developed a manikin respiration system that enables us to visualize and quantify the direction and density of aerosol-laden exhalations of patients being treated with oxygen therapy under prescribed and reproducible conditions. Schematic and image of our experimental apparatus are shown in Fig. 2. A Michigan Instruments dual lung simulator and a ventilator (ParaPAC plus 310) are configured to mimic negative pressure patient respiration with a prescribed tidal volume and frequency. A tube connects the lung simulator with a head simulator module (HSM-A), where the manikin is modified to breathe through the nose and/or mouth. A tidal volume of *V_t_* = 500 ml with a breathing rate of *f* = 12 breaths per minute (bpm) represents normal breathing. Patients that require oxygen assistance typically have a lower tidal volume which is compensated with a higher frequency.^4^ Thus, a tidal volume of *V_t_* = 350 ml and respiratory rate of *f* = 20 bpm simulated a patient with lung disease and model typical COVID-19 patients who are experiencing shortness of breath while undergoing supplemental oxygen therapy.

Metered O_2_ from a pressurized tank is delivered through a nasal cannula (Vyaire Medical Inc.) or a simple O_2_ mask (Vyaire Medical Inc.) with flow rates, *Q*, listed in Table I. Such flow rates are typically used in treating Covid-19 and other respiratory disease patients. All measurements were conducted in a room with heating, ventilation, and air conditioning (HVAC) at 23.5 °C with standard deviation of 0.5 °C, and humidity 21.0% with standard deviation of 2.5%. Such HVAC conditions are within range of standard OSHA regulations, which prescribe that indoor temperatures should remain within 20–24 °C and relative humidity remain within 20%–60%.^33,34^

In order to image the exhalations, we use an aerosol fog composed of approximately 1–5 *μ*m water-based droplets which scatter light while moving with the air flows.^6,14^ While the droplet sizes are optimized for light scattering and are more numerous, they are known to be within the size range of bioaerosols exhaled while breathing.^6,35,36^

Two complementary lighting methods are used to obtain the overall direction and spread of the aerosol-laden exhalations. This helps inform and deduce when the flows are puff-like with linear momentum, vs spreading diffusively as they slow down. We place a 5500 lumen LED white light source behind the head to backlight the aerosols in the exhalations.^12,15^ All of the image analysis is conducted on the backlight videos. Additionally, we use a green laser sheet (532 nm, 40 mW) to visualize the flow in a 2 D plane which helps qualitatively clarify flow structures.^13^ A Pixel 4a smartphone camera is used to capture movies with 1080 p at 30 frames per second (fps) over several exhalation cycles. All quantitative analysis is performed with at least five trials for each set of parameters. Time averages of the exhalations *μ_t_* are obtained as $\mu_{t}=\frac{1}{N}\sum_{t=1}^{N}I(x,y,t)$, where I(x,y,t) is the 2-dimensional image with intensity values corresponding to each *x* and *y* pixel at frame *t*, and *N* is the number of frames.

An aerosol-laden air flow emerging from the nose of the manikin while free-breathing and imaged with laser illumination is shown in Fig. 2(b). The shape of the exhalation puff from the nose and mouth of the manikin is observed to be typical of a fast-moving fluid exiting a nozzle and losing momentum in a quiescent fluid.^37,38^ Tracking the leading edge of an exhalation puff over consecutive frames, we observed nasal exhalations with a speed of 1.21 ± 0.07 m/s (mean ± SD), consistent with the 0.4 to 1.6 m/s range reported for normal human nasal breathing.^36^ These ranges of measured speeds were found to be also consistent with complementary tests conducted with a TES 1341 Hot-Wire Anemometer. The exhalation flow speed is observed to decrease below 0.01 m/s, corresponding to the ambient fluctuations in the room, at about 40–45 cm from the nozzles using this device.

## III. RESULTS

### A. Dispersal through the nasal cannula

**FIG. 3.**
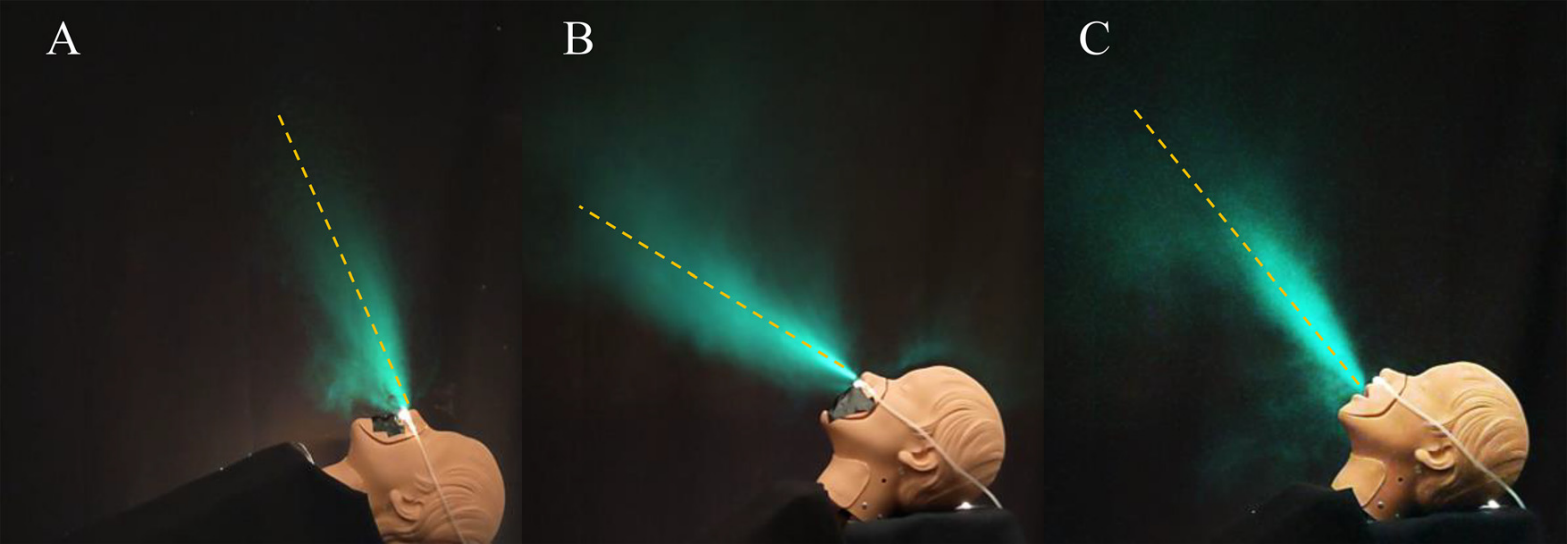
The time-averaged exhalations emerging from the nose of a medical manikin fitted with a nasal cannula when the head rests flat (a), and while it is on a bed propped at 45° (b), and when exhaling through the mouth (c), under shortness of breath conditions (*V _t_* = 350 ml at *f* = 20 bpm; *Q* = 4 lpm). The aerosol-laden exhalation is visualized with back lighting and rendered with artificial cyan coloring. The primary exhalation airflow is observed to spread conically with the principal direction indicated by the dashed line with elevation angle θ=60°±0.5° (a), 19.3° ± 0.5° (b), and 56° ± 1° (c). Multimedia views: https://doi.org/10.1063/5.0057227.1
10.1063/5.0057227.1; https://doi.org/10.1063/5.0057227.2
10.1063/5.0057227.2; https://doi.org/10.1063/5.0057227.3
10.1063/5.0057227.3

Figure 3 shows time-averaged exhalations emerging from the manikin while undergoing oxygenation treatment with a nasal cannula under varying conditions over a time window of five exhalation cycles each (roughly 6 s per exhalation cycle). The corresponding movies can be found in Fig. 3 (Multimedia view). In each case, a primary puff can be observed clearly extending from the nose past the nasal cannula as it enters and spreads conically in the relatively still air in the room while losing speed. A nasal cannula typically only has one major puff coming directly from the nose. But there are examples in which the stem of the nasal cannula may reflect the exhaled air up past the nose and over the head. We observe that the exhalation airflow on average emerges from the nose somewhat similarly in relation to the face, no matter its angle of tilt, i.e., the direction of the exhalation puff is essentially set by the direction of the face, as shown in Figs. 3(a) and 3(b).

While wearing a nasal cannula, a speed of 1.2 ± 0.15 cm/s is observed near the nose, and a distance of 0.35 ± 0.02 m is reached before the exhalation puff loses linear momentum and becomes diffusive under shortness of breath conditions (*Q* = 4 lpm). This is consistent with observations where nasal exhalation puffs extending straight out to about 60 cm have been reported with adult humans that exhale somewhat greater *V_t_* under normal breathing conditions.^36^

When the nose and mouth are both open as in Fig. 3(c), most of the exhalation emerges through the mouth, because of the relatively low resistance offered by the wider and shorter oral passage compared with the nasal passage. The airflow emerges from the mouth at a higher elevation angle of 36° ± 2° in Fig. 3(c) compared with when it emerges from the nose as in Fig. 3(b), under otherwise similar conditions. A greater speed of 1.64 ± 0.08 m/s, and greater distance of 0.51 ± 0.01 m is reached before the exhalation puff becomes diffusive when exhaling through the mouth compared with the nose. Because exhalations through the mouth are at a higher elevation angle, they reach a higher elevation compared to nasal breathing, further increasing the risk to those working near the patient.

**FIG. 4.**
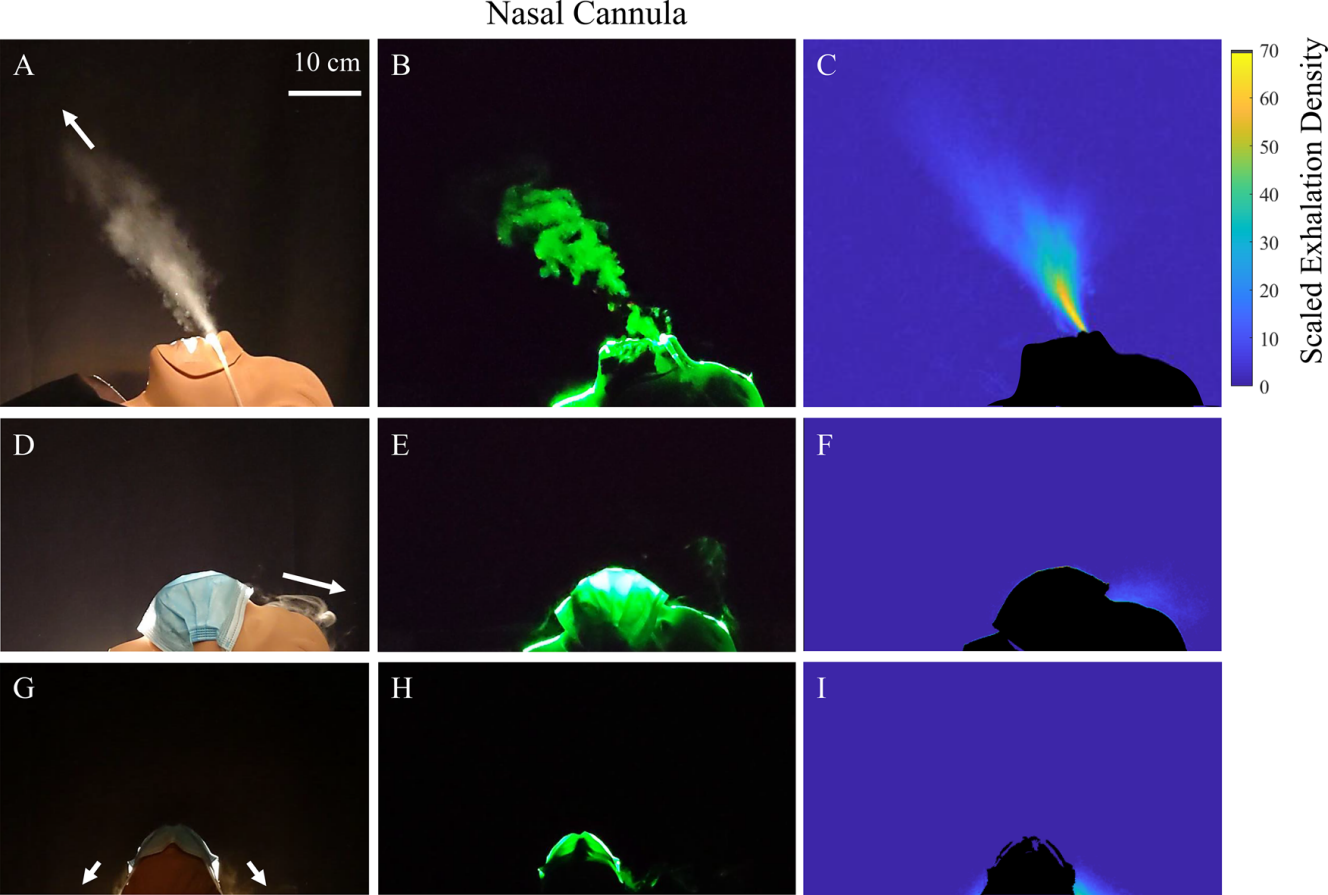
Exhalation puffs emerge from the nose while wearing a nasal cannula with an oxygen delivery flow rate of *Q* = 4 lpm. [(a)–(c)] Saggital view with nasal cannula. [(d)–(f)] Saggital view with cannula and surgical mask on top. Some exhalations are redirected toward the nose bridge of the patient. [(g)–(i)] Transverse view with cannula and surgical mask on top. The exhalation puffs are redirected mostly downward away from the face of a caregiver as indicated by the arrows. The cross-sectional laser illumination in (b) reveals a repeating pattern of swirling vortices signifying considerable linear momentum in the exhalation puffs emerging past the unmitigated nasal cannula (see the corresponding movie). The time-averaged projected exhalation density exhalation scaled by the mean density $\rho_{m}=0.71\times 10^{-3}$ kg/m^2^ if the exhalation were uniform is shown by the color bar. Multimedia views: https://doi.org/10.1063/5.0057227.4
10.1063/5.0057227.4; https://doi.org/10.1063/5.0057227.5
10.1063/5.0057227.5

To illustrate the dynamics, a backlit image of the exhalation puff emerging through the nose past the nasal cannula is shown in Fig. 4(a) (Multimedia view) and the contained vortex dynamics made visible by the cross-sectional laser imaging in Fig. 4(b) (Multimedia view). Here the data corresponding to a midrange of flow rates using *Q* = 4 lpm is shown. To illustrate the corresponding spread of the exhalation near the manikin, Fig. 4(c) shows the corresponding exhalation density averaged over several breath cycles projected in the vertical plane. The exhalation is observed to spread conically forward and concentrate in a single fast-moving main puff as it mixes with the air in the room, loses momentum, and becomes diffusive.

It can be noted that some secondary puffs exist around the nose depending on exactly how the nasal cannula is mounted in the nose. Because of the presence of these puffs, the exhalation density does not decay as rapidly as the inverse square of the distance from the nose/mouth of the patient in all directions in front of the patient. Thus, the puffs end up increasing the concentration of exhalations directly above and in front of the face of a supine patient in a more focused region.

### B. Dispersal through simple O_2_ mask

**FIG. 5.**
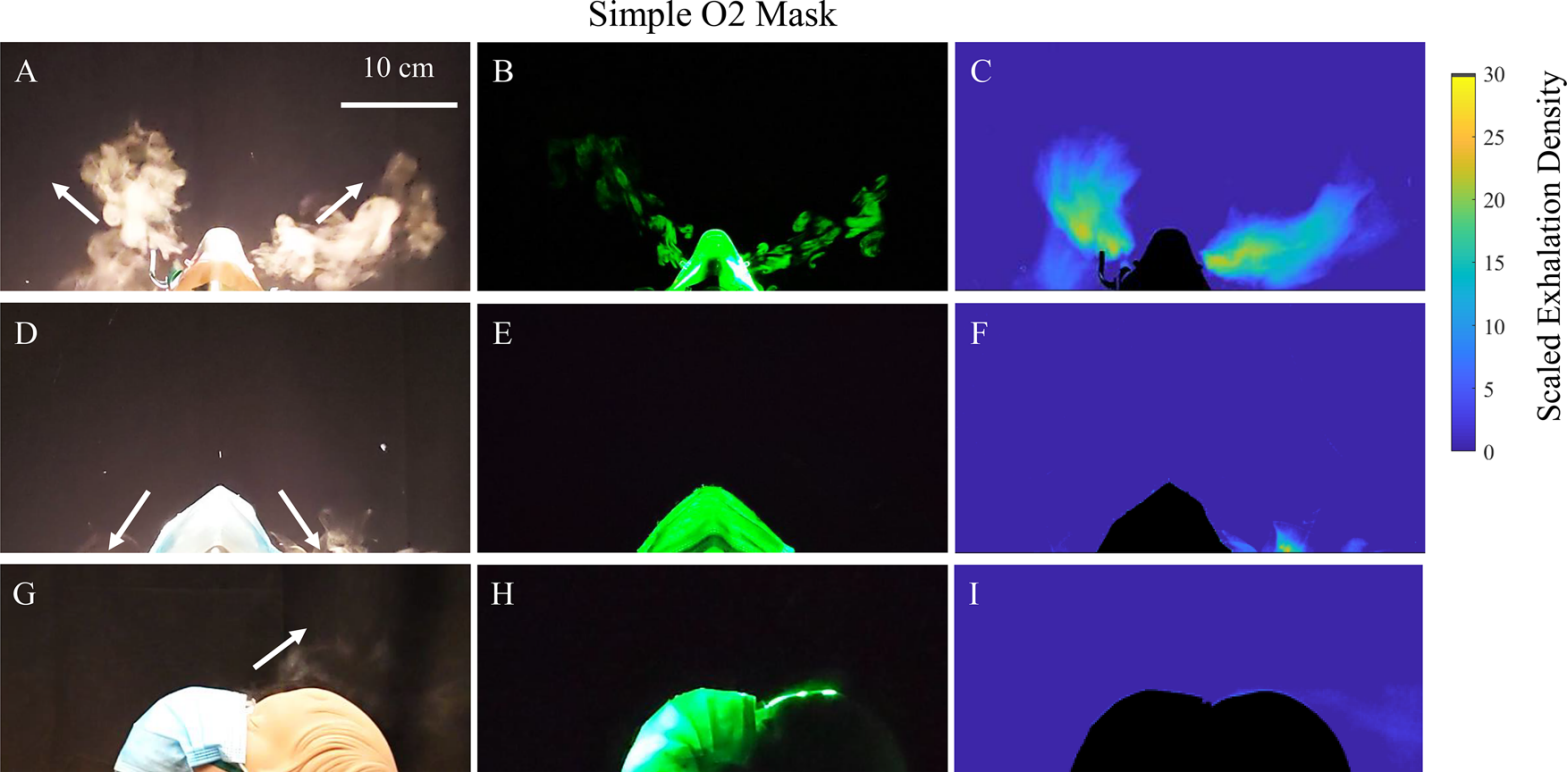
Exhalation puffs emerge from the nose while wearing a simple O_2_ mask with an oxygen delivery flow rate of *Q* = 8 lpm. [(a)–(c)] Transverse view with simple O_2_ mask. [(a)–(f)] Transverse view with simple O_2_ mask and surgical face mask placed on top. With a surgical mask on top, the puffs are redirected mostly downward away from the face of a caregiver as indicated by the arrows. [(g)–(i)] Saggital view with simple O_2_ mask and surgical face mask placed on top. Some of the exhalation also escapes from the nose bridge region. Note the repeating vortex structures along the puff emerging from the simple O_2_ mask vents indicates significant exhalation momentum in (b). The time-averaged projected exhalation density scaled by the mean density $\rho_{m}=1.41\times 10^{-3}$ kg/m^2^ if the exhalation were uniform is shown by the color bar. Multimedia views: https://doi.org/10.1063/5.0057227.6
10.1063/5.0057227.6; https://doi.org/10.1063/5.0057227.7
10.1063/5.0057227.7

A simple O_2_ mask emits three puffs in total, each in different directions. Figures 5(a)–5(c) (Multimedia view) shows that a simple O_2_ mask redirects the exhalations largely through the vents on either side of the device, and to a smaller degree from the gap between the mask and the bridge of the nose. Here the data corresponding to the midrange of flow rates using *Q* = 8 lpm is shown, and the corresponding movies show examples from Figs. 5(a) and 5(b), respectively (Multimedia view). Very little escapes from around the chin because the O_2_ mask fits relatively tightly in that area. The exhalation puffs from the vents on either side of the simple O_2_ mask appear broader and have a rounded shape compared with the puffs coming from the nasal cannula. Vortex structures extending along lines starting at each of the vents are also evident from the cross section laser imaging on either side of the mask in Fig. 5(b), and the associated movie (Multimedia view). Just as in the exhalation past the nasal cannula, these swirling vortices are associated with significant linear momentum when a fast-moving fluid enters a still region. The distance the puffs extend out is approximately 0.29 ± 0.02 m. These slightly lower distances compared to the nasal cannula are consistent with the formation of two dominant puffs vs one dominant puff as in the nasal cannula.

The average exhalation density reached over an entire breathing cycle is shown in Fig. 5(c), looking down from the top of the head, or, the transverse view. While not as elevated as in the case of the nasal cannula, the exhalation density resulting from the puffs emerging from the vents in the simple O_2_ mask is pointed directly where caregivers typically stand while giving care to a supine patient. Because of the presence of the puffs, the exhalation density in the case of the simple O_2_ mask does not decay as rapidly as the inverse of the square of the distance from the face, but rather is concentrated in particular directions. It can be further observed from the color bar that the exhalation densities reached near the vicinity of the head are comparable to those reached near the vicinity of the head while wearing a nasal cannula shown in Fig. 4(c).

Thus, the exhalation density around an unmitigated oxygenation device is greater in different directions in relation to the head because of the presence of the puffs in each device vs if the exhalations were diffusing out uniformly from the device. We further quantify the resulting spatial distribution and concentration levels and effect of oxygenation rates next, before examining the effect of placing a surgical mask.

### C. Time-averaged exhalation cone analysis

**FIG. 6.**
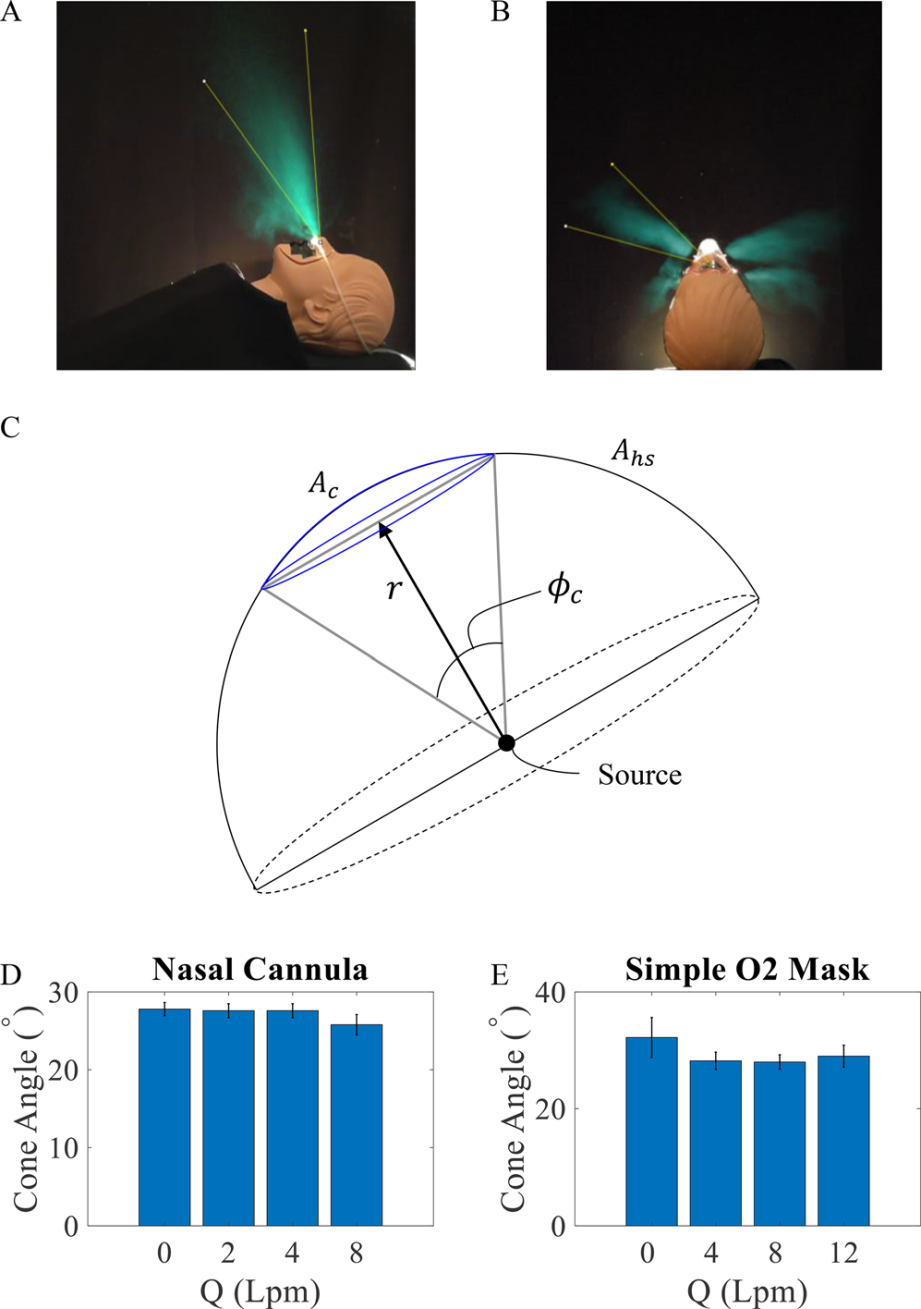
Representative time averages showing the spread of the exhalation puffs in conical regions for the (a) sagittal view of the nasal cannula and (b) transverse view of the simple O_2_ mask. The aerosol-laden exhalation is visualized with back lighting and rendered with artificial cyan coloring. (c) A schematic of the conical exhalation regions with high aerosol concentration relative to a hemisphere around the source. The measured exhalation cone angles $\phi_{c}$ are observed to be essentially constant across the oxygenation flow rates *Q* for (d) nasal cannula and (e) simple O_2_ mask. The data for *Q* = 0 lpm was taken for reference.

When an exhalation puff from the mouth or nose enters the relatively quiescent air, it spreads out in a cone in the time-averaged images as shown in Fig. 6. The Reynolds number, used to characterize the fluid flows, is given by Re $=\frac{DU}{\nu }$, where $\nu =1.6\times 10^{-5}$ m^2^/s is the kinematic viscosity for air, *D* is the jet diameter, and *U* is its speed.^37^ Assuming, D≈2 cm, and U≈1.5 m/s, Re ≈ 2000. The flows corresponding to these Re are considered turbulent, consistent with the observation of vortex swirl patterns seen in the exhalation puffs entering relatively still air while wearing the nasal cannula (Fig. 4, Multimedia views) or the simple O_2_ mask (Fig. 5, Multimedia views). It must be noted here that the observed repeated structures are not simply a result of periodic breaths interacting with each other, but are likely a result of shear instability.^13,19,37,38^ A more in-depth study is needed to confirm this hypothesis.

The measured cone angle $\phi_{c}$ of the turbulent exhalation puffs is shown in Fig. 6. The measured angles are observed to be nearly constant across the oxygenation flow rates, indicating that the cross section of the puffs are not affected by the oxygen flowing from the nasal cannula in the nose. This is also consistent with the near-constant percentage of exhalation observed to move upward and forward as a function of *Q* in the nasal cannula. When a fast-moving jet enters a still fluid from a uniform conduit, there exists a universal value of 23.6° in which the fluid spreads based on the law of similarity.^31,38^ In the case when the manikin is not wearing an oxygen therapy device, the cone angle is approximately 23.5° through the nose and 23.3° through the mouth. The measured cone angles while wearing the nasal cannula are similar to this value with $\phi_{c}=26.4{}^{\circ}\pm 1.5{}^{\circ}$ for nasal breathing, and $\phi_{c}=26.6{}^{\circ}\pm 1.5{}^{\circ}$ for mouth breathing considering the error in measurements. Thus, we deduce that the flow around its stem appears to lead to a slightly larger cone angle compared to the universal value for a jet. By contrast, the observed cone angles $\phi_{c}\approx 29.5{}^{\circ}$ in the case of the simple O_2_ mask are somewhat higher compared with the nasal cannula.

While the time-averaged density of the exhalations decreases uniformly away from the central axis in Fig. 6, one can obtain a simple estimate of the increased risk to exposure assuming that the exhaled aerosols are uniformly distributed within $\phi_{c}$. Then, this focusing of the exhalation puffs in the conical region implies that the concentration of aerosols is given by the ratio of the area of the hemisphere above the patient and the corresponding area of the spherical cap corresponding by $\phi_{c}$ at the same distance *r* from the source [see Fig. 6(c)]. Then, from the area of the spherical cap is $A_{c}=2\pi r^{2}(1-\text{cos}\;(\phi_{c}/2))$, and the area of the hemisphere $A_{hs}=2\pi r^{2}$, we get the relative aerosol concentration ratio $\chi_{r}=1/(1-\text{cos}\;(\phi_{c}/2))$. In the case of the nasal cannula, we accordingly find the concentration ratio *χ_r_* to be approximately 38 times compared to what may be expected if emerging uniformly. In the case of the simple O_2_ mask, we observe that the exhalations emerge mostly in two evenly distributed exhalation puffs. Then, considering the midrange of observed $\phi_{c}\approx 29.5{}^{\circ}$ in the case of the simple O_2_ mask, one can estimate the concentration *χ_r_* to be approximately 15 times higher in the two focused exhalation regions compared with when emerging uniformly in all directions above the face of the patient.

This estimate assumes the density is uniformly distributed in the spherical cap bounded by the conical envelope, whereas the concentrations are even more narrowly peaked around the central axis within the conical region denoted by the arrows in Fig. 6. This approach provides a lower bound for the aerosol concentration near the patient's head. The actual concentrations locally can be even higher in space and time. These measurements and estimates thus highlight the need to mitigate the risk posed by the direct path of the exhalation puffs.

## IV. EXHALED PUFF REDIRECTION WITH SURGICAL MASK COVER

Figures 4(d)–4(i) and 5(d)–5(i) shows the effect of placing a loosely fitted surgical face mask over an oxygenation device under otherwise similar conditions. The term “loosely fitted” refers to the fact that the face mask is clasped behind the manikin ears as designed to be used. During the breathing cycle, no significant deformation in the face mask material was observed, which indicates the lack of a tight sealed fit and indicates ease of breathing. Measuring the pressure in the breathing apparatus, we find no measurable difference whether a surgical mask is placed on, or not, to within measurement fluctuations from breath to breath of ±0.2 cm H_2_O or ±19.6 Pa. Orthogonal views are shown to give a complete picture of the exhalation dispersal with this mitigation strategy. Here, the surgical mask was placed loosely on top of the oxygenation device to limit the effect on the work of breathing.^39^ Thus, the primary objective is to deflect the exhalation puffs, rather than to filter them as in the N95 mask. From these side-by-side images, and the associated movies in Figs. 4 and 5 (Multimedia view) over the range of oxygen flow rates used, we observe that the exhalation puffs are reduced and get deflected behind the manikin face.

Contrasting the unmasked and masked cases in Figs. 4 and 5, the addition of the surgical mask atop the oxygenation devices works to dissipate the initial momentum of the exhaled air, besides redirecting the exhalation puffs coming through the devices downward. Thus, health workers who would otherwise be in the direct pathway of the exhalation when providing care to a supine patient will not face the direct exhalation puff. Rather, the exhalation will be redirected downward to be nearly orthogonal to the directional line between a patient and their caregiver, significantly alleviating the direct exhalation concentration above the mask.

### A. Spatial distribution assessment

The backlit exhalation movies are further analyzed using the MATLAB image processing toolbox. We first conducted background subtraction to isolate the intensity of the illuminated aerosols. The mean measured light intensity, corresponding to the light scattered by the aerosol-laden exhalations after an exhalation cycle, is mapped to the mean projected exhalation density *ρ_m_* in the measured frame encompassing the entire area over which exhalations are observed to reach in one breathing cycle. This mean density is given by the mass of the exhaled volume of air *V_t_* multiplied by the density of exhaled air $\rho_{a}=1.22$ kg/m^3^ and divided by the area of the frame. This density corresponds to the density if the exhalation were uniformly spread and is used to assess the relative risk of the higher dose of virus bearing exhalations in a certain area because of the puffs, in comparison to a scenario in which the exhalations spread out uniformly. The distance of the exhalation puffs is identified by plotting the scaled exhalation density along the observed puffs and then identifying the point where the density has decreased to be within 50% of the mean density.

**FIG. 7.**
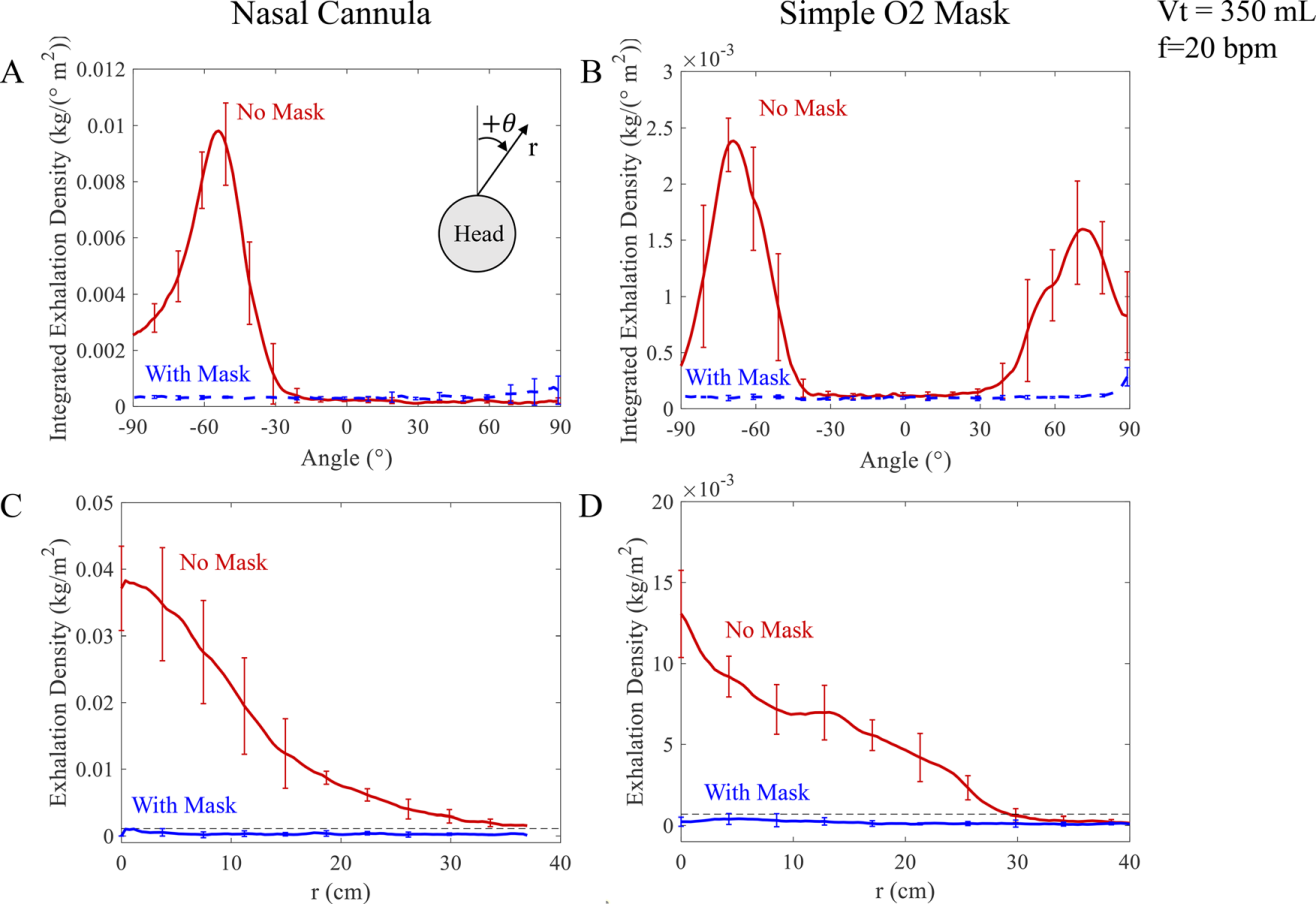
The angular integrated dispersal exhalation intensity is suppressed when a surgical mask is placed on top in both devices [(a) and (b)]. The distance over which the puffs reach is observed to be dramatically curtailed in both devices [(c) and (d)]. The bars represent the range of values among five trials used to calculate the plotted averages. The exhalations travel up to 37 cm for the nasal cannula and 31 cm for the simple O_2_ mask when unmitigated. The black dashed line represents the threshold value that is 50% greater than the mean exhalation density *ρ_m_* used to determine the exhalation distance.

In order to quantify the degree of mitigation, Fig. 7(a) shows the angular exhalation density as a function of angle *θ* around the manikin head as defined in the inset to Fig. 7(a). Here the angular density is obtained by integrating the measured projected exhalation density [as in Figs. 4(c) and 4(f)] from the face out to the furthest distance where exhalations are observed to reach above the face. Both plots corresponding to the exhalations without, and with, the surgical mask fixed atop the oxygenation devices are shown. It can be observed that the large puff which travels upward and forward is clearly suppressed by the placement of the mask. The same angular exhalation density is calculated for a simple O_2_ mask using the views shown in Figs. 5(c) and 5(f) and plotted in Fig. 7(b). Two peaks are observed corresponding to the two principal puffs that emerge upward and outward from the vents of the simple O_2_ mask. As in the nasal cannula, clear suppression of the exhalation puffs is quantitatively observed with the surgical mask placed over the simple O_2_ mask.

To quantify the degree of mitigation with distance above the mask, we plot the exhalation density along the principal puffs in the unmasked cases in the nasal cannula and simple O_2_ mask in Figs. 7(c) and 7(d), respectively. These directions also correspond to the angle at which the maximum in the angular exhalation density occurs in each device in Figs. 7(a) and 7(b). The exhalation density is observed to become significantly lower with the surgical mask on. Comparing the values without and with surgical mask in Figs. 7(c) and 7(d), the exhalation density at *r* = 15 cm can be observed to be at least 30 times smaller in each device. These measurements can be observed to be consistent with the estimates of unmitigated aerosol dispersal in conical regions above the manikin discussed in Sec. III C.

Thus, adding a surgical mask even loosely over either oxygen therapy devices can be seen to quantitatively reduce direct exposure to high exhalation aerosol concentrations created by exhalation puffs above the face.

### B. Dispersal mitigation with oxygen flow rate

**FIG. 8.**
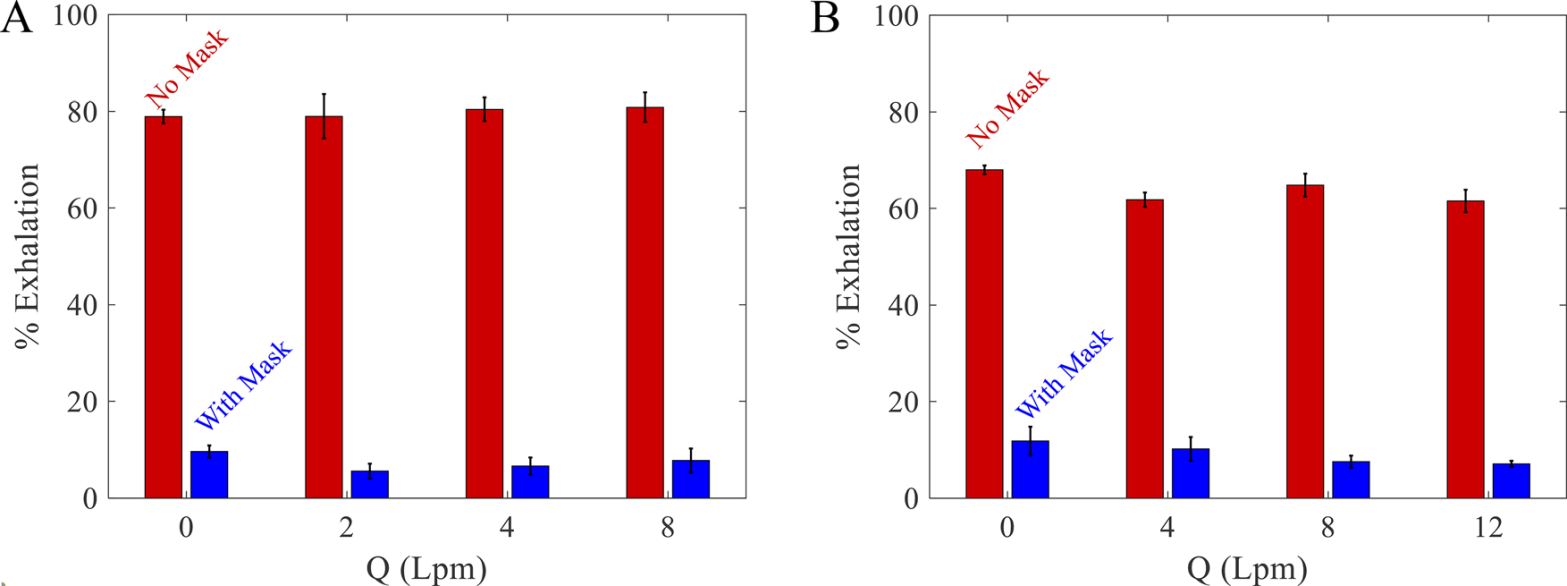
The percent of breath that is exhaled above the face without and with a surgical mask as a function of flow rates for (a) the nasal cannula and (b) the simple O_2_ mask. All cases have five trials.

To quantify the degree of mitigation by a surgical mask with each oxygenation device, the percentage of exhalations observed above the face without and with a surgical mask is obtained by integrating the measured exhalation density above the plane defined by the surgical mask over one exhalation cycle (roughly 6 s). Figures 8(a) and 8(b) show the degree of mitigation observed over the oxygen flow rates examined in the nasal cannula and simple O_2_ mask, respectively, averaged over five independently measured breathing cycles in separate data runs. The percent of exhalations as a function of oxygen flow rates show no particular trend and can be considered more or less flat across the entire range, even while considering the small variations noted by the error bars from the five independent experiments. This is also consistent with the fact that the highly concentrated aerosol conical regions are absent after placement of the surgical mask, as in the nasal cannula exhalation [Figs. 4(c) and 4(f)] and the simple O_2_ mask exhalation [Figs. 5(c) and 5(f)], after placement of the surgical mask.

The average exhalation volume per minute given by the number of breaths per minute times the tidal volume, which is 7 lpm under shortness of breath conditions, and 6 lpm under normal breathing, are similar to *Q* in the case of the nasal cannula. However, if one considers that the majority of the exhalation occurs over a fraction of the breathing cycle, the momentum associated with the exhalation itself can be proportionally higher and can dominate the dispersal over the range of oxygen flow rates used. Using the surgical mask limits the exhalations from the puffs in the directions above the mask to about 10% of the total exhalation volume over the entire range of oxygen flow rates. While exhalations do escape from the sides downward and may diffuse upward (Figs. 4 and 5, second and third rows), this contribution to the exhalation with a surgical mask is significantly less compared with unmitigated exhalations.

Overall, a loosely fitted surgical mask over a nasal cannula, or simple O_2_ mask, redirects exhalations downward and thus away from the faces of caregivers, while simultaneously reducing the volume and density of the exhalations reached above the patient. The surgical mask is only loosely placed to alleviate any concern for increased work of breathing, i.e., to deflect rather than filter the aerosols. The surgical mask is demonstrated to quantitatively reduce exhalation density concentration above the mask. By preventing the exhalations from being launched directly up, the placement of a mask can also suppress wider dispersal of the exhalations depending on the ventilation currents. It should be emphasized that our study pertains to a supine patient and does not apply to patients in the prone position.

## V. CONCLUSIONS

We have constructed a reproducible exhalation system that enables us to quantitatively demonstrate that significant exhalation puff dynamics exist with either a nasal cannula or simple O_2_ mask commonly used in treating COVID-19 and other respiratory disease patients. The exhalations are observed to be concentrated in conical regions in front of the patient as the exhalations move with significant linear momentum before becoming diffusive.

When using the nasal cannula, the exhalations move linearly to significant distances and split slightly around the stem of the device while nasal breathing. The exhalations are angled slightly upwards in comparison to the free-breathing case, and its angle varies somewhat depending on exactly how the nasal cannula is placed. Mouth breathing angles the exhalations relatively higher compared with nasal breathing with a nasal cannula, leading to greater dispersal distances. By contrast, the simple O_2_ mask has upward and lateral puffs that travel out to similar distance from the mask vents on both sides, and one smaller upward puff from the bridge of the nose. However, the exhalations are launched higher and thus spread further as they slowly disperse depending on the airflow within the room. In all cases, the aerosol concentration is found to be many times higher compared to assuming that the exhalations spread uniformly above the patient.

Mitigation is demonstrated by reducing exhalation puffs by using a surgical mask over the oxygenation devices. The mask is loosely placed to redirect rather than filter exhalations. In all cases, the exhalations are directed downward and away from the faces of health care workers working around the patient's head. The surgical mask is found to limit the direct exhalations to about 10% (compared to when no mask is placed) above the face over the entire range of oxygen flow rates used in either device. While the exhaled aerosols spread out over time, this transport is diffusive and can be managed with proper ventilation systems in place. The surgical mask reduces and redirects the exhalations while wearing the nasal cannula and the simple O_2_ mask by dissipating the momentum of the exhalations.

In current practice, there is generally no mitigation in place on the patient side should they sneeze or cough while receiving care. The placement of the mask can also reduce the larger aerosols and droplets expired when the patient speaks, coughs, or sneezes, if not totally eliminate them.^15,35,36,40^ Thus, our study demonstrates the efficacy of placing a surgical face mask over the nasal cannula and the simple O_2_ mask in reducing exhalation exposure risk to caregivers treating patients with COVID-19 and other infectious respiratory diseases.

## Data Availability

The data that support the findings of this study are available within the article.

## References

[1] M. P. Shelly and P. Nightingale, “ Respiratory support,” BMJ 318(7199), 1674–1677 (1999). 10.1136/bmj.318.7199.1674 10373174 PMC1116024

[2] A. S. BaHammam, T. D. Singh, R. Gupta, and S. R. Pandi-Perumal, “ Choosing the proper interface for positive airway pressure therapy in subjects with acute respiratory failure,” Respir. Care 63(2), 227–237 (2018). 10.4187/respcare.05787 29089459

[3] M. Z. Bazant and J. W. M. Bush, “ A guideline to limit indoor airborne transmission of COVID-19,” Proc. Natl. Acad. Sci. 118(17), e2018995118 (2021). 10.1073/pnas.2018995118 33858987 PMC8092463

[4] M. J. Tobin, *Principles and Practice of Mechanical Ventilation* ( McGraw-Hill Education, 2010).

[5] B. R. O'driscoll, L. S. Howard, and A. G. Davison, “ BTS guideline for emergency oxygen use in adult patients,” Thorax 63(Suppl. 6), vi1–vi68 (2008). 10.1136/thx.2008.102947 18838559

[6] D. S. Hui, B. K. Chow, S. S. Ng, L. C. Y. Chu, S. D. Hall, T. Gin, J. J. Y. Sung, and M. T. V. Chan, “ Exhaled air dispersion distances during noninvasive ventilation via different respironics face masks,” Chest 136, 998–1005 (2009). 10.1378/chest.09-0434 19411297 PMC7094372

[7] J. A. McGrath, A. O'Sullivan, G. Bennett, C. O'Toole, M. Joyce, M. A. Byrne, and R. M. MacLoughlin, “ Investigation of the quantity of exhaled aerosols released into the environment during nebulisation,” Pharmaceutics 11, 75 (2019). 10.3390/pharmaceutics11020075 PMC6409895 30759879

[8] D. Brunk MDedge News, “ Noninvasive ventilation: Options and cautions for patients with COVID-19,” CHEST Physician, 21 Sept. 2020; available at www.mdedge.com/chestphysician/article/228771/coronavirus-updates/noninvasive-ventilation-options-and-cautions.

[9] J. B. Fink, S. Ehrmann, J. Li, P. Dailey, P. McKiernan, C. Darquenne, A. R. Martin, B. Rothen-Rutishauser, P. J. Kuehl, S. Häussermann, R. MacLoughlin, G. C. Smaldone, B. Muellinger, T. E. Corcoran, and R. Dhand, “ Reducing aerosol-related risk of transmission in the era of COVID-19: An interim guidance endorsed by the International Society for Aerosols in Medicine,” J. Aerosol Med. Pulm. Drug Delivery 33, 300–304 (2020). 10.1089/jamp.2020.1615 PMC7757542 32783675

[10] C. R. MacIntyre and A. A. Chughtai, “ Facemasks for the prevention of infection in healthcare and community settings,” BMJ 350, h694 (2015). 10.1136/bmj.h694 25858901

[11] D. Tsilingiris, M. Papatheodoridi, and C. J. Kapelios, “ Providing evidence on the ongoing health care workers' mask debate,” Intern. Emerg. Med. 15, 773–777 (2020). 10.1007/s11739-020-02382-4 32468509 PMC7255970

[12] L. Bourouiba, E. Dehandschoewercker, and J. W. M. Bush, “ Violent expiratory events: On coughing and sneezing,” J. Fluid Mech. 745, 537–563 (2014). 10.1017/jfm.2014.88

[13] M. Abkarian, S. Mendez, N. Xue, F. Yang, and H. A. Stone, “ Speech can produce jet-like transport relevant to asymptomatic spreading of virus,” Proc. Natl. Acad. Sci. U. S. A. 117(41), 25237–25245 (2020). 10.1073/pnas.2012156117 32978297 PMC7568291

[14] S. Verma, M. Dhanak, and J. Frankenfield, “ Visualizing the effectiveness of face masks in obstructing respiratory jets,” Phys. Fluids 32(6), 061708 (2020). 10.1063/5.0016018 PMC7327717 32624649

[15] B. Chang, R. S. Sharma, T. Huynh, and A. Kudrolli, “ Aerial mucosalivary droplet dispersal distributions with implications for disease mitigation,” Phys. Rev. Res. 2, 043391 (2020). 10.1103/PhysRevResearch.2.043391

[16] M. Staymates, “ Flow visualization of an N95 respirator with and without an exhalation valve using schlieren imaging and light scattering,” Phys Fluids 32(11), 111703 (2020). 10.1063/5.0031996 PMC7684679 33244212

[17] P. Bahl, C. M. de Silva, A. A. Chughtai, C. R. MacIntyre, and C. Doolan, “ An experimental framework to capture the flow dynamics of droplets expelled by a sneeze,” Exp. Fluids 61(8), 176 (2020). 10.1007/s00348-020-03008-3 32834458 PMC7368616

[18] P. Bahl, S. Bhattacharjee, C. de Silva, A. A. Chughtai, C. Doolan, and C. R. MacIntyre, “ Face coverings and mask to minimise droplet dispersion and aerosolisation: A video case study,” Thorax 75, 1024 (2020). 10.1136/thoraxjnl-2020-215748 32709611

[19] A. Khosronejad, S. Kang, F. Wermelinger, P. Koumoutsakos, and F. Sotiropoulos, “ A computational study of expiratory particle transport and vortex dynamics during breathing with and without face masks,” Phys. Fluids 33(6), 066605 (2021). 10.1063/5.0054204 PMC8188648 34149276

[20] C. S. Ng, K. L. Chong, R. Yang, M. Li, R. Verzicco, and D. Lohse, “ Growth of respiratory droplets in cold and humid air,” Phys. Rev. Fluids 6(5), 054303 (2021). 10.1103/PhysRevFluids.6.054303

[21] K. L. Chong, C. S. Ng, N. Hori, R. Yang, R. Verzicco, and D. Lohse, “ Extended lifetime of respiratory droplets in a turbulent vapor puff and its implications on airborne disease transmission,” Phys. Rev. Lett. 126(3), 034502 (2021). 10.1103/PhysRevLett.126.034502 33543958

[22] L. Wu, X. Liu, F. Yao, and Y. Chen, “ Numerical study of virus transmission through droplets from sneezing in a cafeteria,” Phys. Fluids 33(2), 023311 (2021). 10.1063/5.0040803 PMC7976044 33746490

[23] M. Klompas, M. A. Baker, C. Rhee, R. Tucker, K. Fiumara, D. Griesbach, C. Bennett-Rizzo, H. Salmasian, R. Wang, N. Wheeler, G. R. Gallagher, A. S. Lang, T. Fink, S. Baez, S. Smole, L. Madoff, E. Goralnick, A. Resnick, M. Pearson, K. Britton, J. Sinclair, and C. A. Morris, “ A SARS-CoV-2 cluster in an acute care hospital,” Ann. Intern. Med. 174, 794 (2021). 10.7326/M20-7567 33556277 PMC7924623

[24] J. A. Razzak, J. A. Bhatti, M. R. Tahir, and O. Pasha-Razzak, “ Initial estimates of COVID-19 infections in hospital workers in the united states during the first wave of pandemic,” PLoS One 15(12), e0242589 (2020). 10.1371/journal.pone.0242589 33275599 PMC7717542

[25] See https://vapotherm.com/covid-19/ for information about new practices in respiratory therapy during COVID-19.

[26] J. Li, J. B. Fink, A. A. Elshafei, L. M. Stewart, H. J. Barbian, S. H. Mirza, L. Al-Harthi, D. Vines, and S. Ehrmann, “ Placing a mask on COVID-19 patients during high-flow nasal cannula therapy reduces aerosol particle dispersion,” ERJ Open Res. 7, 00519-2020 (2021). 10.1183/23120541.00519-2020 33527076 PMC7607969

[27] S. H. Cataldo, B. P. Harvey, and M. J. Pedro, “ Comparative efficacy of aerosolized particle filtration by non-invasive ventilation modalities: Simulation of SARS-CoV-19 transmission,” Am. J. Biomed. Sci. Res. 9, 001460 (2020). 10.34297/AJBSR.2020.09.001460

[28] G. E. Mejía-Terrazas and E. López-Muñoz, “ Supplemental oxygen in surgical patients with COVID-19,” J. Anesth. 34(6), 958–958 (2020). 10.1007/s00540-020-02850-3 32862273 PMC7456396

[29] Y. Matsui, T. Takazawa, A. Takemae, and S. Saito, “ Does a surgical mask improve oxygenation in COVID-19 patients?,” JA Clin. Rep. 7(1), 34 (2021). 10.1186/s40981-021-00439-7 33852101 PMC8045437

[30] See A. Khan, W. T. McGee *et al.*, https://www.surveymonkey.com/results/SM-CDRTMHCZ7 for “ Baystate Medical Center National 02 Mask Usage Clinical Care Survey” (last accessed January 23, 2021).

[31] G. N. Abramovich, *General Properties of Turbulent Jets* ( MIT Press, 2003), pp. 3–49.

[32] R. Chou, T. Dana, R. Jungbauer, C. Weeks, and M. S. McDonagh, “ Masks for prevention of respiratory virus infections, including SARS-CoV-2, in health care and community settings,” Ann. Intern. Med. 173(7), 542–555 (2020). 10.7326/M20-3213 32579379 PMC7322812

[33] S. A. Quraishi, L. Berra, and A. Nozari, “ Indoor temperature and relative humidity in hospitals: Workplace considerations during the novel coronavirus pandemic,” Occup. Environ. Med. 77(7), 508–508 (2020). 10.1136/oemed-2020-106653 32424023

[34] R. E. Fairfax, *Reiteration of Existing OSHA Policy on Indoor Air Quality: Office Temperature/Humidity and Environmental Tobacco Smoke—Occupational Safety and Health Administration* ( OSHA, 2003).

[35] J. W. Tang and G. S. Settles, “ Coughing and aerosols,” N. Engl. J. Med. 359(15), e19 (2008). 10.1056/NEJMicm072576 18843121

[36] J. W. Tang, A. D. Nicolle, C. A. Klettner, J. Pantelic, L. Wang, A. B. Suhaimi, A. Y. L. Tan, G. W. X. Ong, R. Su, C. Sekhar, D. D. W. Cheong, and K. W. Tham, “ Airflow dynamics of human jets: Sneezing and breathing—Potential sources of infectious aerosols,” PLoS One 8(4), e59970 (2013). 10.1371/journal.pone.0059970 23560060 PMC3613375

[37] D. J. Tritton, *Physical Fluid Dynamics*, The Modern University in Physics Series ( Springer, The Netherlands, 2012).

[38] B. Cushman-Roisin and J. M. Beckers, *Introduction to Geophysical Fluid Dynamics—Physical and Numerical Aspects* ( Academic Press, 2005).

[39] R. H. Haraf, M. A. Faghy, B. Carlin, and R. A. Josephson, “ The physiological impact of masking is insignificant and should not preclude routine use during daily activities, exercise, and rehabilitation,” J. Cardiopulm. Rehabil. Prev. 41(1), 1–5 (2021). 10.1097/HCR.0000000000000577 33351538 PMC7769052

[40] Z. Y. Han, W. G. Weng, and Q. Y. Huang, “ Characterizations of particle size distribution of the droplets exhaled by sneeze,” J. R. Soc. Interface 10(88), 20130560 (2013). 10.1098/rsif.2013.0560 24026469 PMC3785820

